# Genetic influence on urinary vitamin D binding protein excretion and serum levels: a focus on rs4588 C>A polymorphism in the GC gene

**DOI:** 10.3389/fendo.2023.1281112

**Published:** 2023-12-07

**Authors:** Durmus Doğan, Eda Gül Özcan, Dilek Ülker Çakır, Fatma Sılan

**Affiliations:** ^1^ Department of Pediatric Medicine, Division of Pediatric Endocrinology, Çanakkale Onsekiz Mart University, Çanakkale, Türkiye; ^2^ Department of Pediatric Medicine, Çanakkale Onsekiz Mart University, Çanakkale, Türkiye; ^3^ Department of Biochemistry, Çanakkale Onsekiz Mart University, Çanakkale, Türkiye; ^4^ Department of Medical Genetics, Çanakkale Onsekiz Mart University, Çanakkale, Türkiye

**Keywords:** vitamin D binding protein, polymorphism, Rs4588, serum 25-hydroxyvitamin D, gC gene, urinary excretion

## Abstract

**Introduction:**

Vitamin D binding protein (VDBP) plays a crucial role in vitamin D transport and metabolism. The rs4588-A polymorphism of the GC gene, encoding VDBP, has been associated with altered serum VDBP and 25-hydroxyvitamin D (25OHD) levels. However, the mechanisms underlying these effects remain unclear. We aimed to investigate the relationship between urinary VDBP excretion and serum VDBP and 25OHD levels in individuals with and without the rs4588-A allele.

**Methods:**

A cross-sectional study was conducted on 109 children (mean age: 11.96 years) to explore the impact of rs4588-A on vitamin D metabolism and urinary VDBP excretion. Biochemical analyses determined serum 25OHD and VDBP levels, and urinary VDBP-to-creatinine ratio (u-VDBP/Cr). Genotyping for rs4588 SNP was performed using LightSNiP assay. Statistical analyses included correlation, linear regression, and comparison between allele groups.

**Results:**

Participants carrying the rs4588-A allele exhibited lower serum 25OHD levels compared to non-carriers (median (IQR): 11.85 (3.5) vs. 12.86 (4.9), p = 0.023). However, no statistically significant differences were observed in serum VDBP levels (126.34 ± 59.3 in rs4588-A vs. 136.49 ± 51.3 in non-rs4588-A, p = 0.141) or in u-VDBP/Cr (median (IQR): 0.4 (0.35) in rs4588-A vs. 0.386 (0.43) in non-rs4588-A, p = 0.189) between the two allele groups. A significant inverse correlation between u-VDBP/Cr and serum VDBP levels was found only in rs4588-A carriers (r = -0.367, p = 0.024). No such correlation was observed in non-carriers or the entire cohort. A linear regression analysis confirmed the impact of u-VDBP/Cr on serum VDBP levels in rs4588-A carriers (B = -0.269, t = -2.185, p = 0.035).

**Conclusion:**

Individuals with the rs4588-A allele in the GC gene had lower serum 25OHD levels. An inverse correlation between urinary VDBP excretion and serum VDBP levels was observed, suggesting a partial role of the renal pathway in altered serum VDBP and 25OHD levels linked to the rs4588-A allele.

## 1 Introduction

Vitamin D binding protein (VDBP), originally called GC-globulin, is the primary carrier of vitamin D in circulation. VDBP is encoded by the GC gene located on chromosome 4q12-q13, consisting of 13 exons and 12 introns (1). VDBP is a highly polymorphic protein that has 120 variants that have been demonstrated electrophoretically, and 1242 polymorphisms are listed in the NCBI database. Among these polymorphic alleles, the two most common single nucleotide polymorphisms (SNP) [c.1296T>G encoding D432E (SNPdb rs7041) and c.1307C>A encoding T436K (SNPdb rs4588) are found in exon 11 in complete linkage disequilibrium (1, 2). The haplotype of these nucleotide changes results in the protein isoforms GC1 (GC1f and GC1s) and GC2. The rs7041 SNP results in the reference allele G, while the variant allele is T, resulting in the phenotypic allele GC1s, characterized by glutamic acid (Glu) at position 432 instead of aspartic acid (Asp) as in GC1f. At the rs4588 locus, the reference allele is C and the variant allele is A, resulting in the GC2 phenotypic allele with lysine (K) instead of threonine (T) at position 436. In addition, glycosylation patterns differ between GC1 and GC2; GC1 variants bind to N-acetylgalactosamine at position 436, whereas GC2 lacks this O-glycosylation due to lysine (3).

The effect of these GC SNPs extends to the affinity of VDBP for 25OHD and the subsequent effect on serum levels of VDBP and 25OHD (4–6). Comparative VDBP analyses using different measurement techniques have shown that carriers of the GC2 (rs4588-A allele) genotype have lower VDBP levels (approximately 10%) and 25OHD concentrations (5-15%) compared to GC1 carriers (5–7). Despite these findings, the underlying cause of the lower DBP concentration in GC2 carriers is still not understood (3, 5, 8). The detailed review of VDBP by Boullion et al. notes that it is unknown whether this is due to reduced hepatic synthesis or rapid clearance related to reduced glycosylation status (4).

**Figure 1:**
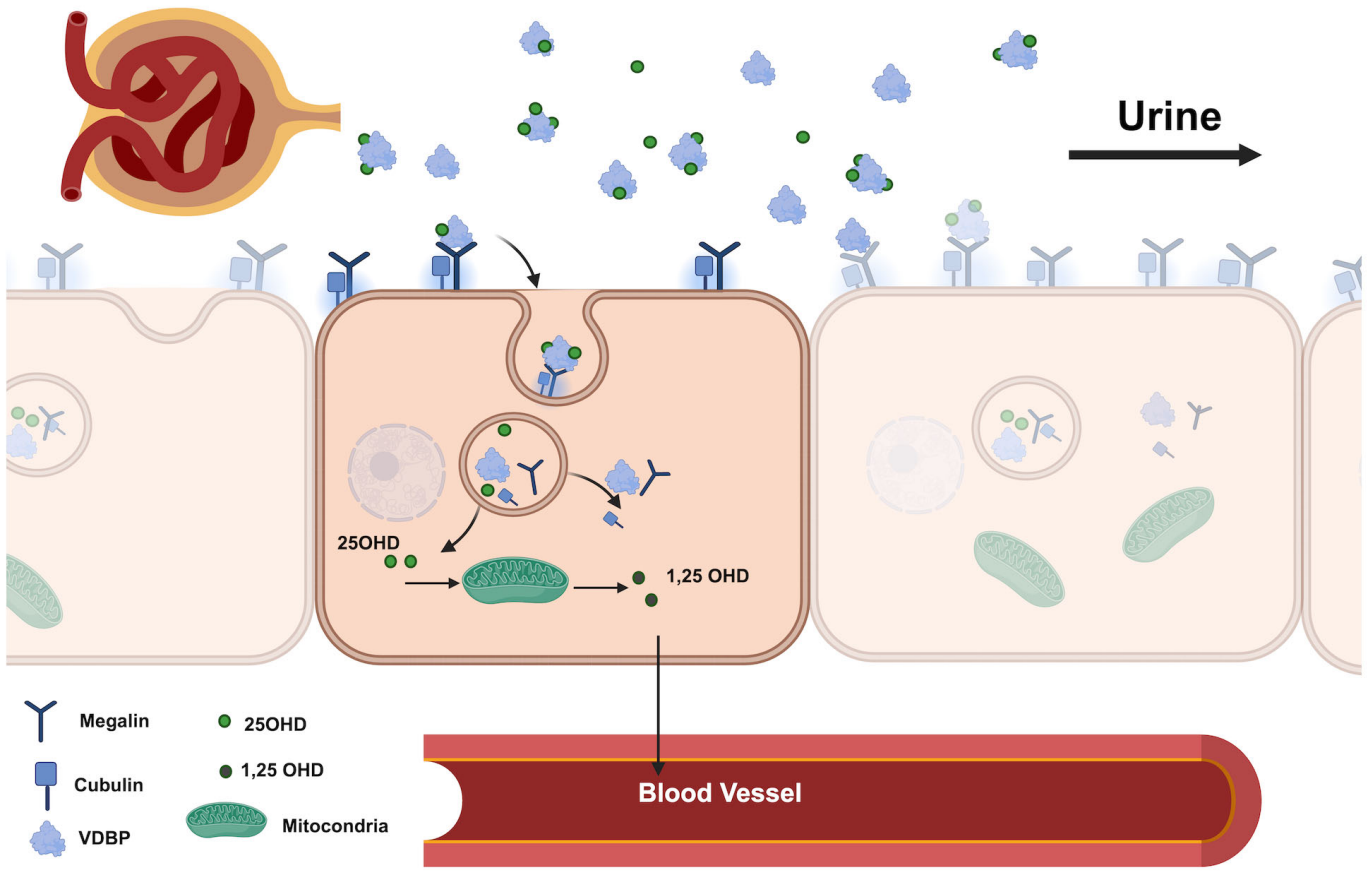
Renal pathway of VDBP and 25OHD in the proximal tubule. (VDBP, Vitamin D Binding Protein; 25OHD, 25-Hydroxyvitamin D; 1.25 OHD, 1.25- dihydroxyvitamin D).

The renal tubular cells are a critical point in the transport of vitamin D via VDBP. VDBP and the VDBP-25OHD holoprotein enter the glomerular filter and are internalized by megalin/cubulin receptors on the apical surface of tubular cells, promoting the formation of 1-25 dihydrocholecalciferol (**Figure 1**) (5). Urinary loss of VDBP, which has a lower molecular weight than albumin, results in urinary excretion of 25OHD and vitamin D metabolites, potentially leading to a poor vitamin D status. Studies by Nykjaer et al. (9) showed excessive urinary loss of VDBP and concomitant 25OHD in megalin knockout mice, and similar conditions were observed in patients with nephrotic syndrome, where poor vitamin D status was associated with urinary loss of VDBP (10). In addition, type 1 diabetic patients with proteinuria may also have low vitamin D status due to urinary VDBP loss (11).

The primary aim of this study was to investigate the variations in urinary VDBP excretion associated with the presence and absence of rs4588-A. Additionally, we aimed to explore the relationship between urinary VDBP levels and serum VDBP and 25OHD levels in individuals with and without the rs4588-A allele. By doing so, our study aims to enhance our understanding of the role of the rs4588-A polymorphism in VDBP regulation and its impact on vitamin D metabolism through the renal tubular pathway. Considering the potential influence of SNPs on receptor-ligand interactions, our findings may also provide valuable insights into the effects of GC gene polymorphisms on the megalin-VDBP interaction at the tubular apical surface.

## 2 Material method

The research was carried out at a tertiary care and referral hospital in the western region of Türkiye. We enrolled healthy boys and girls aged 10-17 years for this cross-sectional study. Exclusion criteria comprised ongoing medication usage, the presence of systemic diseases (such as diabetes mellitus, morbid obesity, metabolic syndrome, chronic kidney disease, epilepsy, etc.), and recent vitamin D supplementation within the preceding 2 months. Postmenarcheal girls underwent evaluation during the follicular phase, ensuring assessment within 10 days of menstruation to maintain cohort homogeneity.

### 2.1 Data and sample collection

To ensure uniformity in measurements, data on height, weight, and puberty assessment were collected for all participants by a single trained physician. After a fasting period of at least 8 hours, approximately 4-5 ml of venous blood was collected from the subjects in the morning. Blood was drawn from the forearm cubital veins into one EDTA (Ethylenediaminetetraacetic acid) tube and a serum separate tube. Simultaneously, approximately 10 ml of spot urine sample was collected from each participant.

### 2.2 Biochemical analysis

All blood samples were collected between 7:30 and 9:30 a.m. and immediately centrifuged to extract serum for analysis (except for whole blood samples used for DNA isolation). The extracted serum samples were stored at -70°C until the date of analysis, while the whole blood samples designated for DNA isolation were stored at -20°C. Biochemical parameters including calcium, phosphorus, creatinine, and alkaline phosphatase (ALP), as well as parathyroid hormone (PTH), were measured using calorimetric methods (Roche-Cobas 6000 and Roche-Cobas 8000 instruments). Serum 25OHD levels were determined using the competitive inhibition enzyme immunoassay technique. Serum VDBP levels were also measured by enzyme-linked immunosorbent assay (ELISA) with a commercially available VDBP kit (ELK Biotechnology Wuhan, China). Urine creatinine levels were also determined using the appropriate kit procedure and urine VDBP levels were determined according to the kit procedure (ELK Biotechnology Wuhan, China). Vitamin D deficiency was defined as a serum 25OHD level of < 12 ng/ml (12).

### 2.3 Calculation of bioavailable and free 25OHD

Free 25OHD and bioavailable 25OHD values were calculated by using the results of serum VDBP, albumin, and total 25OHD vitamin using mathematical formulae previously reported in the literature (13). A free 25OHD ratio was obtained by proportioning free 25OHD to a total 25OHD. The binding coefficient of VDBP with 25OHD was taken as 6x10^5^ M for the 25OHD binding coefficient of 7x10^8^ M albumin. The molecular weight of albumin was 66430 da and 58000 da for VDBP.

### 2.4 Genetic analyses

Genomic DNA was extracted from peripheral blood lymphocytes on the day of analysis using the EZ1&2DNA blood 200µlKit (QIAGEN) kit. Genotypes of the rs4588 SNP of the GC gene were determined using the LightSNiP assay, which utilizes the polymerase chain reaction (PCR) process. In each 20-microliter PCR reaction tube, we added 10.4 microliters of H2O, 1 microliter of reagent mix, 5 microliters of DNA, 1.6 microliters of MgCl2 (25mM), and FastStart DNA Master 2.0 microliter, following the protocol: an initial denaturation step at 95°C for 10 minutes, followed by denaturation at 95°C for 15 seconds, annealing at 60°C for 1 minute, extension at 72°C for 1 minute, and a final extension at 72°C for 10 minutes. DNA concentration measurements were performed using a NanoDrop spectrophotometer, with a concentration range maintained between 50-100 ng/μl for PCR. For the rs4588 SNP, we used the following primer sequences: the forward primer 5’-AGGCTTGGGAAGAGTGCTTT-3’ and the reverse primer 5’-GTGCTTTGGCGTGAAGGTTT-3’. The genotyping procedure involved the use of simple probes (LightSNiP, TibMolBiol) and the LightCycler FastStart DNA Master HybProbe Kit (Cat. No. 12239272001, Roche), in combination with the LightCycler 480 Instrument II (Roche) for PCR analysis. Genotyping was based on a melting curve-based analysis method.

### 2.5 Statistical analysis

Demographic characteristics were summarized using mean age, gender distribution, and pubertal status. Continuous variables (serum 25OHD levels, VDBP levels, u-VDBP/Cr ratio) were presented as mean (SD) or median (IQR), and their normality was assessed using the Kolmogorov-Smirnov test. Categorical variables (prevalence of vitamin D deficiency, gender, pubertal status) were expressed as percentages. Allele and genotype frequencies were calculated to explore genetic variations. Comparative analyses assessed intergroup differences, employing independent t-tests or Mann-Whitney U tests for continuous variables and Chi-square for categorical variables (p < 0.05).

Correlation analysis evaluated relationships using Pearson or Spearman coefficients, visualized via scatter plots for patterns. Logarithmic transformation (log10) normalized data, exploring u-VDBP/Cr impact on serum VDBP levels and potential nonlinear trends. Cook’s distance identified influential data points. Linear regression assessed log-transformed u-VDBP/Cr and serum VDBP correlation within allele groups. Statistical analysis utilized SPSS (IBM Corp., Version 23.0, Armonk, NY, USA), with GraphPad Prism 9 (GraphPad Software, Boston, USA) for graphical representation as appropriate.

### 2.6 Sample size

In this study, we aimed to examine the relationship between urinary VDBP and serum VDBP levels in individuals carrying the rs4588-A allele. Given the lack of similar studies and available data, we conducted a sample size calculation to ensure an adequate number of participants with the rs4588-A allele. For this, we used the G*Power software, taking into account the allele frequencies reported in previous studies in Türkiye (14, 15). This calculation factored in important parameters including a significance level (α) of 0.05, a statistical power (1-β) of 0.95, and the anticipated proportions of rs4588-C and rs4588-A allele carriers based on prior studies conducted in Turkey. The resulting calculated sample size for each allele group was 42, leading to a total sample size of 84 participants. Recognizing the significance of obtaining robust and meaningful outcomes, we aimed to include between 110-115 participants, a number aligned with Kücükali et al.’s study (15), to establish a strong and well-founded statistical basis for our findings.

## 3 Result

### 3.1 Study population characteristics

**Figure 2:**
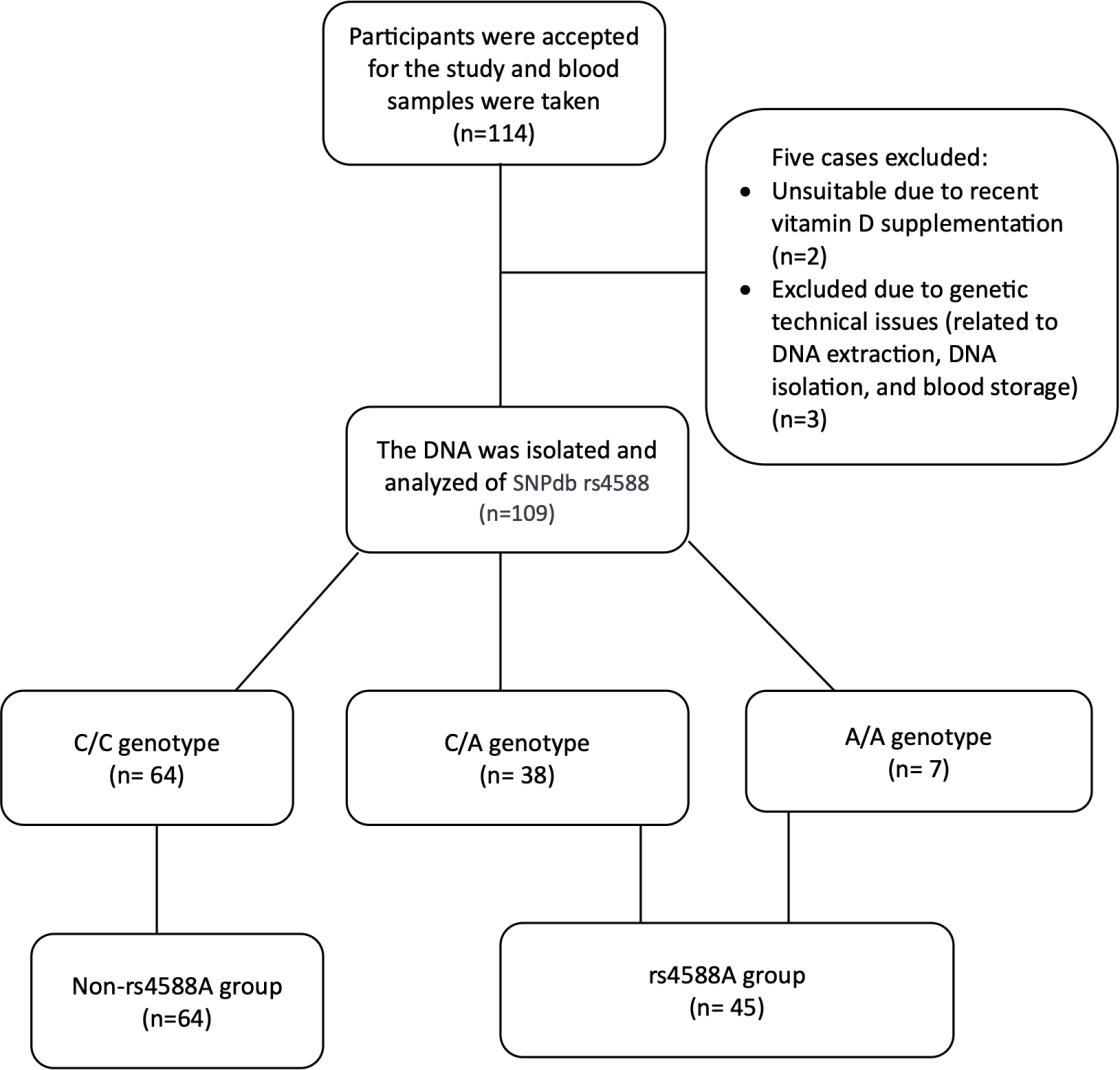
Flow chart of the cross-sectional study to compare participant distribution based on rs4588 genotype.

A total of 114 cases admitted to pediatric outpatient clinics were initially included. However, following blood sample collection, five cases were subsequently excluded: two due to recent vitamin D supplementation and three due to genetic technical issues related to DNA extraction, DNA isolation, and blood storage. As a result, a total of 109 children, with an average age of 11.96 years, remained as participants in this study (**Figure 2**). The study population characteristics are summarized in **Table 1**. Among the participants, 63.3% (n = 69) were female, and 73.4% (n = 80) were in the pubertal period. The median (IQR) serum 25OHD level was measured at 12.57 (4.62) ng/ml (**Table 1**). And a notable 72.5% of the cases were identified as vitamin D deficient. Examination of parameters related to 25OHD and VDBP, including bioavailable 25OHD, serum VDBP, and u-VDBP/Cr, revealed no significant differences based on gender or pubertal status (**Table 2**).

**Table 1:** Anthropometric and laboratory characteristics of the study participants.

Variables	All subjects
Female, n (%)	69 (63)
Prepubertal, n (%)	22 (21)
Age (years)	12.13 (5.44)
Height (Z-score)	0.076 ± 1
BMI (Z-score)	0.37 ± 1.42
Calcium (mg/dl)	9.76 ± 0.57
Phosphorous (mg/dl)	4.43 ± 0.82
ALP (IU/L)	182 (164)
PTH (pg/ml)	43.9 (26.8)
VDBP ug/ml	132.36 (88)
25OHD (ng/ml)	12.57 (4.62)
u-VDBP/Cr	0.398 (0.38)
% free 25OHD	0.051 (0.03)
Bioavailable 25OHD (ng/ml)	2.94 (1.83)

Values are presented as median (IQR) and mean ± SD. IQR: interquartile range, SD, standard deviation; ALP, alkaline phosphatase; PTH, parathyroid hormone; VDBP, vitamin D-binding protein; 25OHD, 25-hydroxyvitamin D; u-VDBP/Cr, urinary VDBP-to-creatinine ratio; BMI, Body mass index. Height and BMI are presented as z-scores adjusted for age and gender, calculated based on Turkish child growth standards.

**Table 2:** Analysis of vitamin D and related parameters based on gender and pubertal characteristics^*^.

Characteristic	25OHD	Bioavailable 25OHD	VDBP	u-VDBP/Cr
Gender				
Male	12.6 (5.11)	2.98 (1.56)	144.7 (87.2)	0.404 (0.32)
Female	12.6 (5.36)	2.95 (2.25)	120.6 (87.1)	0.353 (0.4)
Pubertal Status				
Prepubertal	12.86 (7.2)	2.95 (2.19)	136 (101.6)	0.419 (0.19)
Pubertal	12.57 (4.68)	2.95 (1.84)	131.2 (87.1)	0.373 (0.41)

Values are presented as Median (IQR). ^*^Statistical tests for differences between groups were performed, and p-values > 0.05 indicate non-significant differences. VDBP, Vitamin D-binding protein (presented in ug/ml); 25OHD, 25-hydroxyvitamin D (presented in ng/ml); u-VDBP/Cr, urinary VDBP-to-creatinine ratio. Bioavailable 25OHD presented with ng/ml.

### 3.2 Allele frequencies and genotypic analysis

In our study, we observed allele frequencies of 0.76 for rs4588-C and 0.23 for rs4588-A. The frequency of individuals with the non-rs4588-A allele, all of whom had the rs4588 (CC) genotype, was 0.58. Genotype frequencies for (CC), (CA), and (AA) were 0.587, 0.348, and 0.064, respectively (**Table 3**). Importantly, the distribution of rs4588 genotypes and alleles conformed to the Hardy-Weinberg equilibrium (p = 0.734).

**Table 3:** Allele and Gene frequencies of rs4588 polymorphisms among the cohort^*^.

Alleles (n = 220)	Allele	Frequency
	C	0.766
	A	0.233
Genotypes (n =110)	Genotype	Frequency
	CC	0.587
	CA	0.348
	AA	0.064

^*^The distribution of rs4588 genotypes and alleles conform to Hardy-Weinberg equilibrium (p = 0.734).

**Figure 3:**
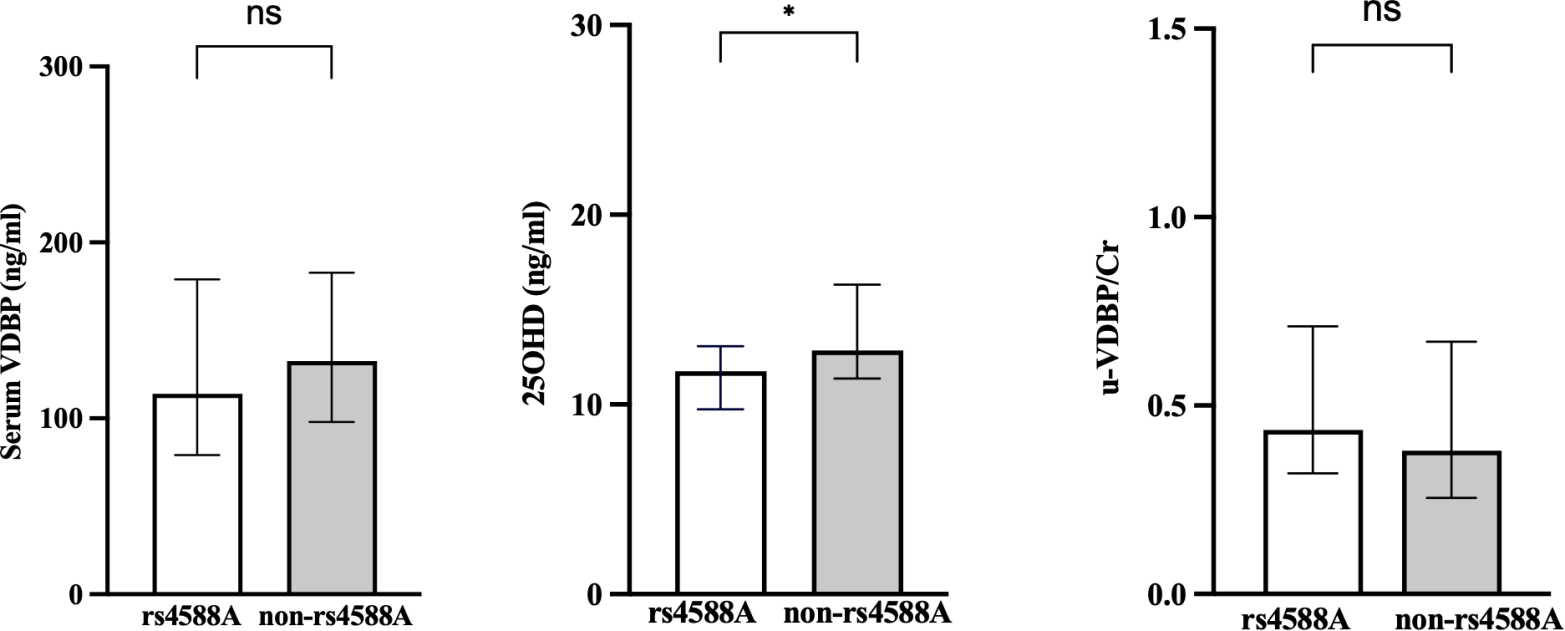
Comparison of the rs4588-A and non-rs4588-A groups in terms of serum VDBP, serum 25OHD, and urinary VDBP/Cr. VDBP, vitamin D-binding protein; 25OHD, 25-hydroxyvitamin D; u-VDBP/Cr, urinary VDBP-to-creatinine ratio. ns, not significant. *, p < 0.05.

The serum 25OHD values were significantly lower in the rs4588-A allele group compared to the non-rs4588-A group (**Figure 3**). The median (IQR) value of serum 25OHD was 11.85 (3.5) in the rs4588-A group and 12.86 (4.9) in the non-rs4588-A group (p = 0.023). Although the serum VDBP was lower and u-VDBP/Cr was higher in the rs4588-A allele group, these differences were not statistically significant for serum VDBP (126.34 ± 59.3 in rs4588-A vs. 136.49 ± 51.3 in non-rs4588-A, p = 0.141) and for u-VDBP/Cr (median (IQR): 0.41(0.35) in rs4588-A vs. 0.386 (0.43) in non-rs4588-A, p = 0.189) (**Figure 3**). Other parameters were not different between these two groups (**Table 4**). The prevalence of vitamin D deficiency was found to be 76% in the rs4588-A allele group and 68% in the non-rs4588-A allele group. Notably, this difference was not statistically significant (X^2 =^ 0.539, p = 0.463).

**Table 4:** Comparison of age, gender, and laboratory findings between rs4588-A and non-rs4588-A groups.

Variables	rs4588-A	non-rs4588-A	P
Female, n (%)	28 (66.7)	40 (60)	0.525
Prepubertal, n (%)	11 (26)	11 (16.7)	0.231
Age (years)	12.01 (5.72)	12.33 (4.73)	0.294
Height (Z-score)	0.302 (0.95)	-0.67 (1.02)	0.075
BMI (Z-score)	0.433 ± 1.36	0.328 ± 1.47	0.747
Calcium (mg/dl)	9.72 ± 0.45	9.78 ± 0.39	0.455
Phosphorous (mg/dl)	4.51 ± 0,69	4.39 ± 0.67	0.261
ALP (IU/L)	202.5 (168)	179.5 (143)	0.449
PTH (pg/ml)	43.93 (29.68)	43.95 (25.02)	0.862
%free 25OHD	0.056 (0.03)	0.049 (0.02)	0.130
Bioavailable 25OHD (ng/ml) (IQR)	2.95 (1.71)	2.91 (1.97)	0.748

Values are presented as median (IQR) and mean ± SD. IQR, interquartile range; SD, standard deviation; ALP, alkaline phosphatase; PTH, parathyroid hormone; VDBP, vitamin D-binding protein; 25OHD, 25-hydroxyvitamin D; u-VDBP/Cr, urinary VDBP-to-creatinine ratio. BMI, Body mass index. Height and BMI are presented as z-scores adjusted for age and gender, calculated based on Turkish child growth standards.

### 3.3 Correlation analysis

#### 3.3.1 Entire group

**Figure 4:**
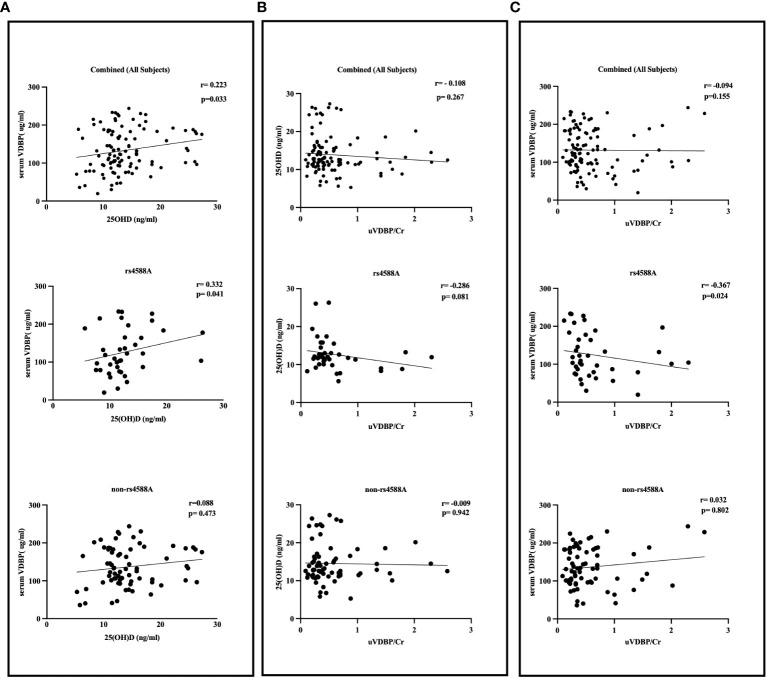
Correlation between serum VDBP, serum 25OHD, and urinary VDBP/Cr in rs4588-A, non-rs4588-A, and combined (all subjects) groups. **(A)** Correlation between serum VDBP and serum 25OHD, **(B)** Correlation between serum 25OHD and urinary VDBP/Cr, **(C)** Correlation between serum VDBP and urinary VDBP/Cr. VDBP: vitamin D-binding protein, 25OHD: 25-hydroxyvitamin D, u-VDBP/Cr: urinary VDBP-to-creatinine ratio.

No significant correlations were detected between VDBP and either age or BMI nor between 25OHD and either age or BMI in the entire study group. No significant correlations were found between u-VDBP/Cr and serum VDBP (r = -0.094, p = 0.155), or between u-VDBP/Cr and 25OHD (r = -0.108, p = 0.267) for the entire cohort (**Figure 4**). However, a significant positive correlation was identified solely between serum VDBP and 25OHD levels (r = 0.223, p = 0.033).

#### 3.3.2 rs4588-A and non-rs4588-A subgroups

In the rs4588-A allele group, we observed significant inverse correlations between u-VDBP/Cr and serum VDBP levels (r = -0.367, p = 0.024) and between serum VDBP and 25OHD levels (r = 0.332, p = 0.041) (**Figure 4**). However, within this group, there was no significant correlation between u-VDBP/Cr and 25OHD levels (r = -0.286, p = 0.081). In contrast, these correlations were not observed in the non-rs4588-A allele group (u-VDBP/Cr vs serum VDBP r = 0.032, p = 0.802; serum VDBP and 25OHD r = 0.088, p = 0.473; u-VDBP/Cr and 25OHD r = -0.009, p = 0.802). Our linear regression analysis demonstrated a significant impact of u-VDBP/Cr on serum VDBP levels within the rs4588 (A) allele group (B = -0.269, t = -2.185, p = 0.035).

## 4 Discussion

This study investigated the relationship between urinary excreted VDBP, serum 25OHD, and serum VDBP, with a particular focus on the rs4588 C>A allele, a well-known SNP recognized for its influence on vitamin D and serum VDBP concentrations. Individuals carrying the rs4588-A allele exhibited lower serum 25OHD levels compared to those without this genetic variation. However, no significant difference was observed in serum VDBP levels between these groups. Notably, a significant inverse correlation emerged between urinary VDBP/Cr and serum VDBP levels in subjects with this minor allele change. This correlation was absent in individuals lacking the rs4588-A allele, as well as in the entire cohort. These findings suggest that the rs4588-A allele may exert an effect on vitamin D metabolism and serum VDBP levels via the renal tubular pathway.

The frequency of the rs4588-A allele, as reported in the NCBI 1000 Genomes Project Phase 3 sequence database, varies across populations, with rates of 0.24 in Europe, approximately 0.06 in African populations, and 0.20 globally (16). Our study’s allele frequency data align with European rates, indicating similarity in allele distribution. Despite limitations in local cohort sizes, our observations are consistent with prior studies in our country, reinforcing the robustness of our findings (14, 15).

GWAS studies have highlighted the significant effects of various GC polymorphisms in the vitamin D pathway (6, 17–19). Consistent with these results, we observed significantly lower 25OHD levels in participants carrying the rs4588-A allele in our study, similar to many other studies. The polymorphism encoding GC2 (with the rs4588-A variation defining this phenotype variant) results in 5-15% lower 25OHD levels compared to the polymorphism encoding GC1 (which carries the C allele in rs4588) (5). This relationship suggests that GC polymorphisms may contribute to lower serum VDBP levels, particularly in individuals with GC2/GC2 homozygosity, potentially resulting in reduced 25OHD concentrations (4). Although we observed lower VDBP levels in participants with the rs4588-A allele in our study, the difference was not statistically significant. This was because the presence of the A allele at rs4588 constituted a relatively small cohort. This may have affected significance.

The relationship between serum VDBP levels and GC polymorphisms has yielded inconsistent findings over time. Initially, studies noted disparities in VDBP values among different racial and ethnic groups, which were initially linked to reduced serum 25OHD levels while free 25OHD levels appeared unaffected (20). However, subsequent studies unveiled a crucial factor contributing to these discrepancies: the limitations of specific monoclonal assays (4). These assays failed to detect specific GC1f haplotypes, leading to artificially lower VDBP measurements. It’s worth noting that not all monoclonal tests displayed the same bias error (4).

Interestingly, some monoclonal studies reported consistent VDBP levels even when GC1f was present (21, 22). Our study contributes to this discourse by employing a monoclonal test, which, in line with previous research, revealed 8% lower VDBP levels in GC2 cases. According to researchers, genotype has a minor impact on serum VDBP levels- resulting in 8-10% lower VDBP even with modern measurement methods employed (4). However, currently, there is no literature explaining the underlying causes of the observed differences in 25OHD and VDBP levels, especially in individuals with the GC2 variant. Our study embarks on an exploration of a potential mechanism, primarily within the renal pathway, which may elucidate these observed variations.

Our findings demonstrated a positive correlation between serum VDBP and serum 25OHD across the entire cohort, as well as within the rs4588-A allele subgroup. This correlation was not evident within the non-rs4588-A subgroup that contained the “CC” allele. Augmenting our results, Carpenter et al. identified a similar positive correlation in a substantial cohort of infants and young children (23). Notably, the GC2 phenotype has been associated with lower VDBP and 25OHD levels. Furthermore, Ballond et al. discovered a positive correlation between serum 25OHD and serum VDBP among both male and female adults (24). These findings align with the correlations observed in our entire cohort and within the rs4588-A subgroup. However, studies involving cohorts with diabetes and CKD have not demonstrated this association, highlighting variability in the relationship across differing health conditions (25, 26). We did not observe this association within the non-rs4588-A group. This finding is intriguing as there is currently no comparable study with the healthy population available for reference. Variations in the distribution of GC genotypes within populations that have been examined for the relationship between VDBP and 25OHD may influence the existence of this association.

We did not observe a significant correlation between u-VDBP/Cr and serum 25OHD, neither in the entire group nor in sub-groups. We could not find an adequate study to compare our results. Kim et al. have reported an absence of association between 25OHD and u-VDBP in a small cohort composed of individuals with type 1 diabetes and controls (27).

In the non-rs4588-A group and the entire group, we were unable to detect a correlation between serum VDBP and u-VDBP/Cr. The available literature does not present studies that offer a basis for comparison with the healthy population regarding this research’s findings. In studies of heart failure and diabetic complications, Elisa et al. and Abdellan et al. found no link between serum VDBP and u-VDBP (28, 29). Conversely, Fayzn et al. observed a positive correlation between u-VDBP, serum VDBP, and urinary albumin in type 2 diabetes (30). It is known that there is a positive correlation between proteinuria and urinary VDBP excretion (31). However, studies on patients with nephrotic syndrome have shown conflicting effects of urinary VDBP excretion on serum VDBP levels (10, 32). These contradictory results in the literature may be associated with the inclusion of different patient groups; nonetheless, the distribution of GC polymorphisms has not been examined in these patient groups.

The lack of observed correlation between serum and urinary VDBP levels in the entire cohort and in the non-rs4588-A subgroup, suggests that the reuptake process mediated by megalin/cubulin may not be associated with serum VDBP, indicating that VDBP may undergo a robust reuptake mechanism. In renal pathologies, this relationship could be disrupted, potentially leading to increased urinary VDBP excretion. However, the impact of GC polymorphisms on urinary VDBP excretion remains unexplored, both in healthy individuals and in disease models. It might suggest that in individuals with the non-rs4588-A variant, serum VDBP levels are influenced by other factors that were not identified or measured in this study. These could include different rates of VDBP synthesis and metabolic clearance that are not solely dependent on renal reabsorption.

Remarkably, our study revealed a significant inverse correlation between u-VDBP/Cr ratio and serum VDBP levels specifically in individuals carrying the GC2 variant linked to the rs4588-A allele. This genetic variation, characterized by the replacement of threonine (T) with lysine (K) at position 436, results in disrupted glycosylation, a process with profound implications. Glycosylation plays a pivotal role in modifying protein structure, function, and interactions, including those with receptors (33). In the context of VDBP, glycosylation likely mediates its interaction with megalin receptors within renal tubules. In GC2 carriers, this interaction might impair VDBP reabsorption, elevating urinary VDBP excretion and partially reducing serum VDBP levels. As serum VDBP tightly binds vitamin D metabolites, this modified interaction could indirectly influence serum 25OHD levels. This offers a plausible explanation for the observed correlation between urinary excreted VDBP and serum VDBP levels in rs4588-A allele carriers, warranting further investigation.

Understanding these complex genetic influences on vitamin D homeostasis may improve our clinical approach to patients who have difficulty optimizing vitamin D levels. Our findings suggest that genetic variations in megalin and ligand interactions, particularly within the renal tubule, may play an influential role in vitamin D metabolism. This information reveals the potential for personalized approaches to vitamin D therapy when these polymorphisms are present. Moreover, what we learn from the complex interplay of megalin, VDBP and rs4588 may contribute to our understanding of protein interactions within the renal pathway (such as the megalin-albumin interaction).

### 4.1 Future studies

Future studies should aim to elucidate specific interactions between megalin/cubulin receptors and VDBP or VDBP-25OHD holoprotein, especially in the context of GC polymorphisms. Understanding these interactions at the molecular level will provide valuable insights into the mechanisms underlying the observed effects on vitamin D metabolism and serum VDBP levels. It will also be of interest to consider possible alterations due to GC polymorphisms in other tissues (parathyroid, placenta, etc.) where megalin-mediated vitamin D uptake is observed. While the impact of SNPs and glycosylation on interaction and protein structure alteration is recognized, the involvement of the intronic polymorphism rs22822679 in the GC gene, which does not directly affect VDBP structure, yet correlates with reduced 25OHD and VDBP levels, offers a compelling area for further investigation. Understanding these complex genetic influences on vitamin D homeostasis can enhance our clinical approach to patients with difficulties in optimizing vitamin D levels.

### 4.2 Limitations

While our study provides information on the association between urinary VDBP excretion, serum VDBP levels, and the rs4588-A allele, several limitations should be noted. Our study’s population specificity calls for caution when generalizing these findings to other ethnic or geographic groups. The cross-sectional study design precludes the establishment of causal relationships and temporal dynamics between variables.

We acknowledge the potential for bias introduced by the use of a monoclonal assay for VDBP measurements, given limitations in detecting certain genetic variants. However, our results align with advanced techniques, bolstering their reliability. Additionally, while we focused on the renal tubule pathway, other factors contributing to vitamin D dynamics, such as dietary intake, sunlight exposure, and other genetic polymorphisms, were not comprehensively addressed. The lack of direct assessment of megalin/cubulin receptor interactions is a notable limitation. Investigating these interactions at the molecular level may provide more comprehensive insights into the underlying mechanisms.

## 5 Conclusion

In summary, individuals carrying the rs4588-A allele in the GC gene exhibit lower serum 25OHD levels, and we uncovered a significant inverse correlation between urinary VDBP excretion and serum VDBP levels. These findings suggest a potential, though partial, role of the renal pathway in explaining altered serum VDBP and 25OHD levels in the presence of the rs4588-A allele. Further research is essential to elucidate the intricate mechanisms at play in individuals carrying this allele and their implications for vitamin D metabolism.

## Data availability statement

The raw data supporting the conclusions of this article will be made available by the authors, without undue reservation.

## Ethics statement

The studies involving humans were approved by Çanakkale Onsekiz Mart University Clinical Research Ethics Committee. The studies were conducted in accordance with the local legislation and institutional requirements. Written informed consent for participation in this study was provided by the participants’ legal guardians/next of kin.

## Author contributions

DD: Conceptualization, Methodology, Writing – original draft. EÖ: Data curation, Project administration, Writing – original draft. DÇ: Formal Analysis, Writing – review & editing. FS: Formal Analysis, Supervision, Writing – review & editing.
