# Supplementary figures and images for: Genetic influence on urinary vitamin D binding protein excretion and serum levels: a focus on rs4588 C>A polymorphism in the GC gene
Source: Front Endocrinol (Lausanne). 2023 Dec 7;14:1281112. doi: 10.3389/fendo.2023.1281112 (PMC10740204; doi:10.3389/fendo.2023.1281112)

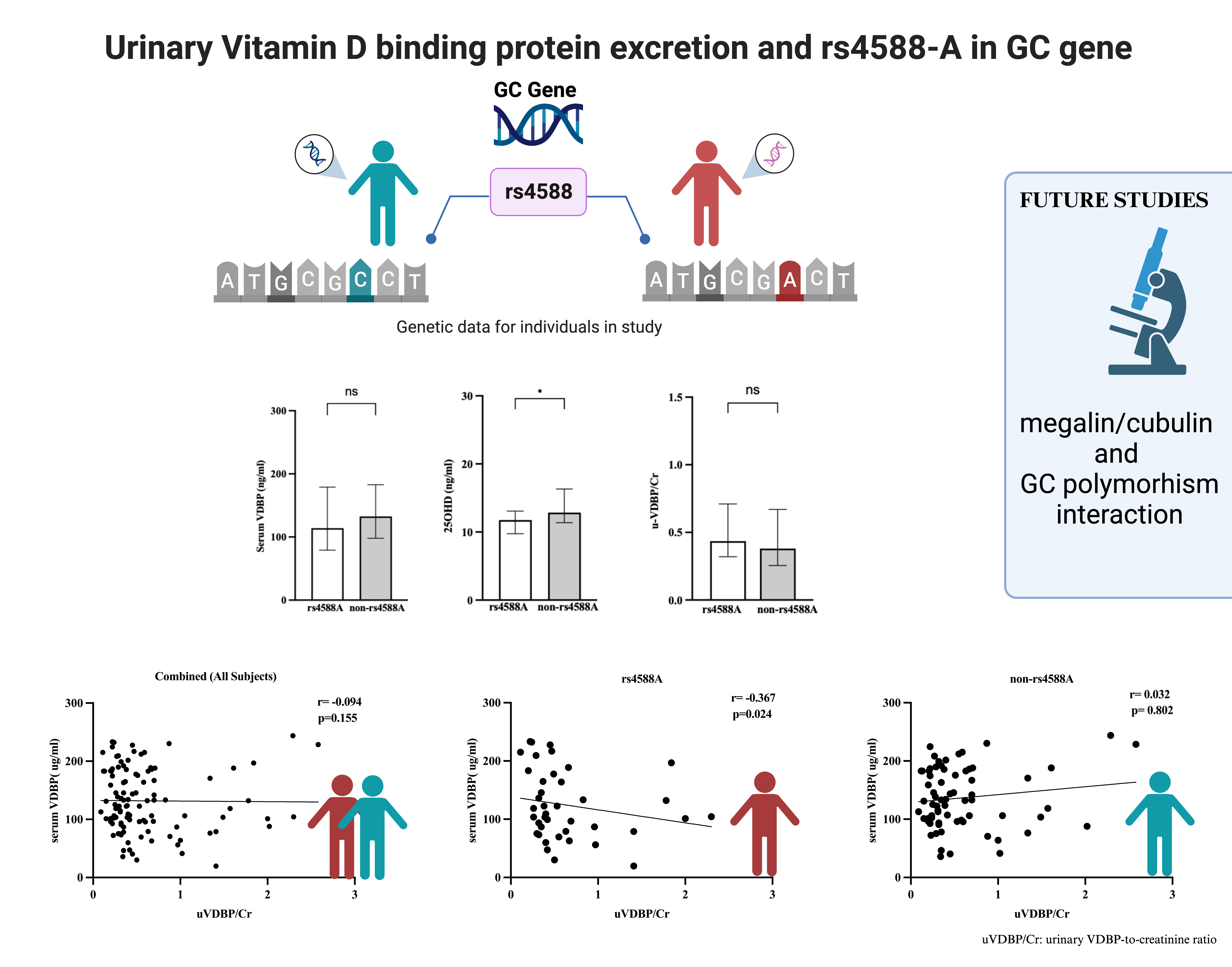

Supplement: Supplementary file 1 [file Image_1.jpeg]
